# Construct Validity and Confirmatory Factor Analysis of the National Center on Health, Physical Activity and Disability Wellness Assessment Tool

**DOI:** 10.3390/healthcare14081074

**Published:** 2026-04-17

**Authors:** Tanjila Nawshin, Navneet Kaur Baidwan, Hui-Ju Young, James Rimmer, Tapan Mehta

**Affiliations:** 1 Department of Health Services Administration, School of Health Professions, University of Alabama at Birmingham, Birmingham, AL 35233, USA; 2 Department of Family and Community Medicine, Heersink School of Medicine, University of Alabama at Birmingham, Birmingham, AL 35294, USA; nbaidwan@uabmc.edu (N.K.B.); tapanmehta@uabmc.edu (T.M.); 3 National Center on Health, Physical Activity and Disability (NCHPAD), Birmingham, AL 35209, USA; hjyoung@uab.edu (H.-J.Y.); jrimmer@uab.edu (J.R.); 4 School of Health Professions Research Collaborative, University of Alabama at Birmingham, Birmingham, AL 35233, USA; 5 Center for Engagement in Disability Health and Rehabilitation Sciences (CEDHARS), Birmingham, AL 35209, USA; 6 Department of Occupational Therapy, School of Health Professions, University of Alabama at Birmingham, Birmingham, AL 35233, USA

**Keywords:** physical, mental, emotional, spiritual, wellness, disability, validity

## Abstract

**Highlights:**

**What are the main findings?**

- The mental and emotional/spiritual wellness domains of the National Center on Health, Physical Activity and Disability (NCHPAD) Wellness Assessment (NWA) tool showed strong convergent validity, and all domains showed strong divergent validity.
- Confirmatory factor analysis confirmed appropriateness of a three-factor model for the NWA tool.

**What are the implications of the main findings?**

- By establishing the NWA as a reliable and practical tool, rehabilitation professionals, health educators and health coaches will gain access to a structured framework for assessing and monitoring key wellness domains.
- Having a brief validated wellness tool ensures a smoother transition from clinical care to self-directed health management, empowering individuals with disabilities to maintain and improve their physical, mental and emotional/spiritual well-being.

**Abstract:**

**Background/Objectives**: To evaluate construct (convergent and divergent) validity and conduct confirmatory factor analysis (CFA) of the National Center on Health, Physical Activity and Disability (NCHPAD) Wellness Assessment (NWA) tool. **Methods**: A cross-sectional survey validation study utilizing secondary data. We assessed Spearman correlations between NWA and 36-Item Short Form Health Survey (SF-36), NWA and Godin Leisure-Time Exercise Questionnaire (GLTEQ) and NWA and Modified Fatigue Impact Scale (MFIS) scores to determine construct validity. A CFA was conducted to test the appropriateness of a three-factor model for NWA. **Results**: Data from 149 participants were used to assess construct validity and from 180 participants for CFA. Both correlations between NWA mental wellness domain and SF-36 mental component scores and between NWA emotional/spiritual wellness domain and SF-36 emotional well-being scores were 0.61 (*p* < 0.001 for both). The correlation between NWA physical wellness domain and SF-36 physical component score was −0.06 (*p =* 0.45). The correlations of NWA with GLTEQ overall and with health contribution scores were 0.26 and 0.30, respectively (*p* < 0.001 for both). The correlations of all NWA domain and MFIS subscale scores ranged between −0.42 and −0.25 (*p* < 0.05). The CFA model’s comparative fit index was 0.90. **Conclusions**: The NWA physical wellness domain did not demonstrate strong convergent validity, as mental and emotional/spiritual wellness domains did. All domains showed strong divergent validity, and CFA showed evidence supporting a three-factor model. Future efforts will emphasize refining and reevaluating the physical wellness domain until it achieves strong psychometric properties.

## 1. Introduction

The available literature supports the effectiveness of behavioral interventions such as exercise, nutrition and mindfulness in promoting health and well-being [1]. However, people with physical disability (PD) transitioning out of rehabilitation often struggle to independently manage health, highlighting the need to integrate strategies into their recovery to enhance wellness.

There is a lack of valid, reliable and succinct tools to monitor wellness behaviors and outcomes in people with PD. Short, simple tools that assess holistic wellness [2]—including exercise, nutrition, mindfulness, sleep and stress management—could greatly benefit rehabilitation professionals in monitoring physical and psychosocial health. Existing tools that assess wellness are excessively lengthy and impractical for clinical or research settings [3]. The wellness evaluation of lifestyle (WEL) tool assesses wellness using 123 items [4,5]. Revised versions of WEL such as the Five-factor WEL (91 items) and Four-factor WEL (56 items) remain burdensome. A similar burden exists for the optimal living profile (OLP) [6], Body–Mind–Spirit Wellness Behavior and Characteristic Inventory (BMS-WBCI) [7,8] and Perceived Wellness Survey (PWS) instruments [9]. Given that health promotion programs and research studies often require participants to complete multiple surveys, longer surveys may introduce a significant response burden [10]. Moreover, none of these tools have been specifically validated for individuals with PD.

To address the need for a brief wellness assessment tool for individuals with PD in real-world settings, the National Center on Health, Physical Activity and Disability (NCHPAD) developed the 16-item NCHPAD Wellness Assessment (NWA) tool, formerly the University of Alabama at Birmingham (UAB)/Lakeshore Wellness Assessment (LWA) [11]. The NWA evaluates physical, mental, and emotional/spiritual wellness domains of health for participants in NCHPAD’s Mindfulness, Exercise and Nutrition to Optimize Resilience (MENTOR) program [11]. Developed through collaboration between disability experts and researchers, the tool was initially assessed for face validity [11]. However, its construct validity and confirmatory factor analysis (CFA) were not assessed.

At this initial phase of NWA tool development, our primary objective was to evaluate how effectively it measured the physical, mental and emotional/spiritual wellness constructs using an existing dataset of individuals with PD and to assess whether the current structure of NWA requires modification. Assessing construct validity and conducting confirmatory factor analysis (CFA) was essential for examining the tool’s internal structure and dimensional alignment. Therefore, the purpose of this study was to assess the construct validity of NWA and identify potential areas for improvement in how it measures physical, mental and emotional/spiritual wellness in individuals with PD.

## 2. Materials and Methods

### 2.1. Study Design

The study was a cross-sectional secondary data analysis [12]. Baseline data from MENTOR participants between February–November 2022 were analyzed to assess the validity of NWA [11,13]. Additionally, an extended analysis was conducted using data from participants recruited between January 2023–August 2024 to ensure the consistency of the results. Details about the MENTOR program are described elsewhere [11].

### 2.2. Participants

Participants were recruited across the United States from rehabilitation centers, local communities, a commercial entity that sells rehabilitation equipment and social media advertisements. The eligibility criteria were as follows: (1) aged 18–90 years, (2) self-reported physical/mobility disability, (3) access to internet and (4) reside in the United States. This project was part of evaluation activities under NCHPAD, designated and approved on 20 December 2021, by the University of Alabama at Birmingham (UAB) institutional review board as a quality improvement project (IRB-300008580).

### 2.3. Study Tools

Both convergent and divergent validity of NWA were evaluated by examining its correlations with other validated instruments used in MENTOR. Convergent validity was assessed by comparing NWA with 36 item Short Form Health Survey (SF-36) and Godin Leisure-Time Exercise Questionnaire (GLTEQ) scores. In the extended analysis, NWA was compared with Patient-Reported Outcomes Measurement Information System (PROMIS) Global-10 scores to assess convergent validity, as SF-36 and GLTEQ were not administered to this cohort. Divergent validity was assessed by comparing NWA with Modified Fatigue Impact Scale (MFIS) scores. A brief description of each instrument used in the analysis is provided below.

#### 2.3.1. National Center on Health, Physical Activity and Disability (NCHPAD) Wellness Assessment (NWA)

The NWA is a 16-item wellness assessment covering physical, mental and emotional/spiritual domains [11]. Each domain includes five items; the 16th item assesses overall wellness. Each item is rated on a 1–5 Likert scale, where 1 indicates very unsatisfied and 5 indicates very satisfied. Domain scores are calculated by summing up the item scores, resulting in a range of 5–25 for each domain. The overall wellness item score is multiplied by 5 to get a range from 5 (very unsatisfied) to 25 (very satisfied). The total score is calculated by summing the domain and overall wellness scores, which yields a range of 20–100. Higher scores indicate better outcomes.

#### 2.3.2. 36-Item Short Form Health Survey (SF-36)

The SF-36 is a 36-item tool with eight health domains: physical functioning, pain, emotional well-being, role limitations due to physical health and emotional problems, energy/fatigue, social functioning and general health [14,15]. The SF-36 physical component score (PCS) and mental component score (MCS) were calculated following Farivar’s 2007 article [16]. The SF-36 was chosen as a comparator for assessing convergent validity because of its good domain alignment with the Wheel of Wellness (WoW) model, which is an established integrative model for wellness [15,17].

#### 2.3.3. Godin Leisure-Time Exercise Questionnaire (GLTEQ)

The GLTEQ is a three-item tool quantifying time invested in vigorous, moderate and light intensity physical activity over a 7-day period [18]. Its intraclass correlation coefficient (ICC) for test-retest reliability is 0.98 [19]. The GLTEQ was chosen as a comparator to assess the convergent validity of NWA’s physical wellness domain because physical activity has a significant association with physical health [20].

#### 2.3.4. Patient-Reported Outcomes Measurement Information System Global Health-10 (PROMIS Global-10)

The PROMIS Global-10 is a 10-item tool for assessing physical and mental health across chronic diseases [21]. It has a Cronbach’s alpha of 0.91–0.99, and test-retest reliability correlation of 0.725–0.883 measured in people with rheumatoid arthritis [22]. The PROMIS-10 was chosen as a comparator for assessing convergent validity because it reliably measures key wellness components such as physical and mental health.

#### 2.3.5. Modified Fatigue Impact Scale (MFIS)

The MFIS is a 21-item tool that measures the effect of fatigue on an individual’s physical, cognitive and psychosocial functioning [23]. The MFIS was chosen as a comparator for assessing divergent validity because it has high (>0.7) internal consistency (Cronbach’s alpha: ≥0.93) [24] and test-retest reliability (ICC: 0.85) [25] measured in people with multiple sclerosis. Although it estimates fatigue, which has some degree of conceptual overlap with wellness, we chose MFIS to assess divergent validity of NWA because MFIS was the only available tool in the MENTOR dataset that measured a construct furthest away from wellness.

### 2.4. Statistical Analysis

#### 2.4.1. Descriptive Statistics

Descriptive statistics were reported as mean ± SD for continuous variables and as frequency (%) for categorical variables, including participant demographics and scores from NWA, SF-36, GLTEQ and MFIS.

#### 2.4.2. Convergent Validity

Convergent validity of NWA’s physical wellness domain was reported using Spearman correlation analysis with physical functioning, pain and physical component scores (PCS) of SF-36, GLTEQ overall and health contribution scores and PROMIS global physical health T-scores in the extended analysis. Convergent validity of NWA’s mental wellness domain was reported using Spearman correlation analysis with mental component scores (MCS) of SF-36 and PROMIS global mental health T-scores in the extended analysis. The convergent validity of NWA’s emotional/spiritual wellness domain was reported using the emotional well-being scores of SF-36. We hypothesized that NWA will show strong convergent validity when compared with these tools (i.e., Spearman correlation ≥ 0.5) [26].

#### 2.4.3. Divergent Validity

The divergent validity of all domains of NWA was assessed using Spearman correlation analysis with all MFIS subscales. We hypothesized that NWA will demonstrate divergent validity when compared with MFIS, as indicated by weak Spearman correlations (i.e., Spearman correlation between >−0.5 and <0.5) [26].

#### 2.4.4. Confirmatory Factor Analysis

A CFA using the maximum likelihood estimation (MLE) method was conducted to test the appropriateness of a three-factor NWA model developed by the researchers and disability experts. The MLE method was used because the observed variables followed a multivariate normal distribution [27]. Model fit was evaluated using several fit indices and was considered a good fit if it had an insignificant chi-square statistic (χ2), comparative fit index (CFI) ≥ 0.90 or a root mean square error of approximation (RMSEA) ≤0.05 with a pclose test value >0.05 [28]. We hypothesized that CFA will provide evidence for the appropriateness of a three-factor NWA model.

Additionally, the internal consistency reliability of the NWA was estimated using Cronbach’s α, with a value of ≥0.70 suggesting acceptable internal consistency [29]. Observations with missing data were excluded from the analysis after observing the pattern of missingness and determining whether missingness was completely at random using the Little’s MCAR test [30]. Results were considered significant at α < 0.05. We performed all analyses using Stata version 18 (Serial Number: 401809313730; StataCorp LLC, College Station, TX, USA).

## 3. Results

### 3.1. Descriptive Statistics

#### 3.1.1. Participant Demographics

The mean (±SD) participant age was 51.76 ± 14.67 years, with 68.33% female, 61.67% white and 23.89% having brain-related primary disabilities. Demographic details are presented in Table 1.

#### 3.1.2. Instrument Score Summaries

A total of 180 participants with disabilities completed all the NWA items, while 22 participants had missing data on at least one item. There was no underlying systematic pattern of missingness and a statistical test determined that missingness was completely at random (Little’s MCAR test was not significant, chi-square statistic (12) = 10.44, *p* = 0.58). Among 180 participants, 149 completed both the SF-36 and MFIS. GLTEQ overall and health contribution scores were available for 172 and 174 participants, respectively. NWA score summaries are reported in Table 2; other instrument score summaries are presented in Appendix A (Table A1).

### 3.2. Convergent Validity

#### 3.2.1. Correlations Between NWA and SF-36

NWA Physical Wellness: All correlations between the NWA physical wellness and SF-36 physical functioning items ranged from −0.16–0.11, with none reaching statistical significance. Correlations between NWA physical wellness items and SF-36 PCS showed similar patterns.

When comparing NWA physical wellness with the SF-36 pain items, the NWA items on satisfaction with sleep and managing physical pain showed positive and significant correlations (Spearman correlations: 0.28 and 0.43, respectively, *p* < 0.001 for both) with the SF-36 item on bodily pain. However, no correlations were ≥0.5.

NWA Mental Wellness: All items within the NWA mental wellness domain correlated positively (ranging from 0.38–0.53) and significantly (*p* < 0.001 for all) with the SF-36 MCS score. The correlation between the NWA mental wellness domain and SF-36 MCS was 0.61 (*p* < 0.001).

**Figure 1:**
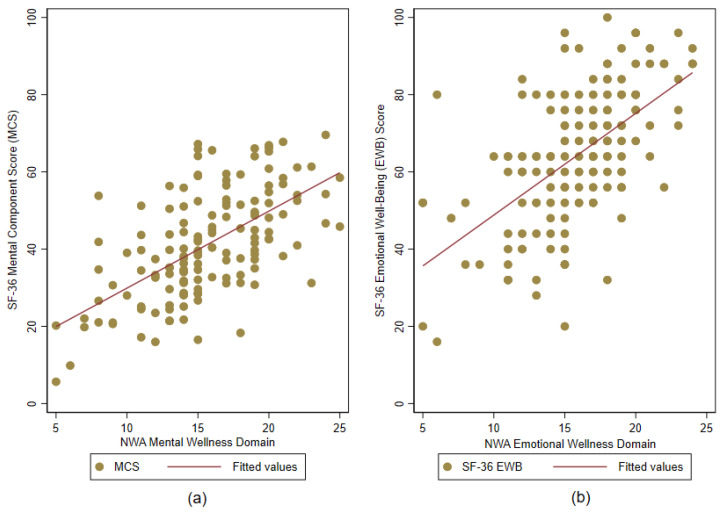
Convergent validity of National Center on Health, Physical Activity and Disability (NCHPAD) Wellness Assessment (NWA) mental and emotional/spiritual wellness domains (N = 149): (**a**) Short Form-36 (SF-36) mental component scores plotted against NWA mental wellness domain scores; (**b**) SF-36 emotional well-being scores plotted against NWA emotional/spiritual wellness domain scores.

NWA Emotional/Spiritual Wellness: All correlations between the NWA emotional/spiritual wellness items and SF-36 emotional well-being domain score were positive (ranging from 0.30 to 0.56, *p* < 0.001 for all). The correlation between the NWA emotional/spiritual wellness domain and SF-36 emotional well-being domain was 0.61 (*p* < 0.001). Figure 1 shows the domain-level correlations of NWA mental and emotional/spiritual wellness scores with SF-36 MCS and emotional well-being scores, respectively.

NWA Overall and Total Wellness: Both NWA overall and total scores showed significant positive correlations with the SF-36 MCS (Spearman correlations: 0.50 and 0.62, respectively; *p* < 0.001 for both) and emotional well-being scores (Spearman correlations: 0.59 and 0.66, respectively; *p* < 0.001 for both). Table 3 shows the Spearman correlations between NWA and SF-36 domains. The item level correlations between NWA and SF-36 are presented in the Supplementary Material (Tables S1–S3).

#### 3.2.2. Correlations Between NWA and GLTEQ

The item level correlations between NWA physical wellness and GLTEQ overall scores ranged between −0.01 to 0.31; and the item level correlations between NWA physical wellness and GLTEQ health contribution scores ranged between 0.01 to 0.40. The correlations were highest and significant (*p* < 0.001) for the NWA item on regular physical exercise with both GLTEQ scores. The Spearman correlations between NWA and GLTEQ are reported in Appendix A (Table A2).

#### 3.2.3. Correlations Between NWA and PROMIS Global-10

Correlations between NWA physical wellness items and PROMIS global physical health ranged from 0.23–0.39 (*p* < 0.001 for all). The item-level correlations between NWA mental wellness and PROMIS global mental health ranged from 0.41–0.55, with a correlation of 0.60 at domain level (*p* < 0.001 for all). The results of this extended analysis are included in the Supplementary Material (Tables S4–S6).

### 3.3. Divergent Validity

NWA Physical Wellness: NWA items showed weak and negative correlations with all the MFIS physical, cognitive, and psychosocial subscale items. The domain score had weak correlations (ranging from −0.40 to −0.25, *p* < 0.05 for all) with all MFIS composite scores.

NWA Mental Wellness: NWA items had weak and negative correlations with the majority of the MFIS physical and psychosocial subscale items, and all the MFIS cognitive subscale items. Most of these correlations were statistically significant (*p* < 0.05). The domain score also had weak correlations (ranging from −0.42 to −0.26, *p* < 0.05) with all MFIS composite scores.

NWA Emotional/Spiritual Wellness: NWA items had weak and negative correlations with the majority of the MFIS physical and psychosocial subscale items, and all the MFIS cognitive subscale items. The domain score also had weak correlations (ranging from −0.38 to −0.29, *p* < 0.001) with all MFIS composite scores.

**Figure 2:**
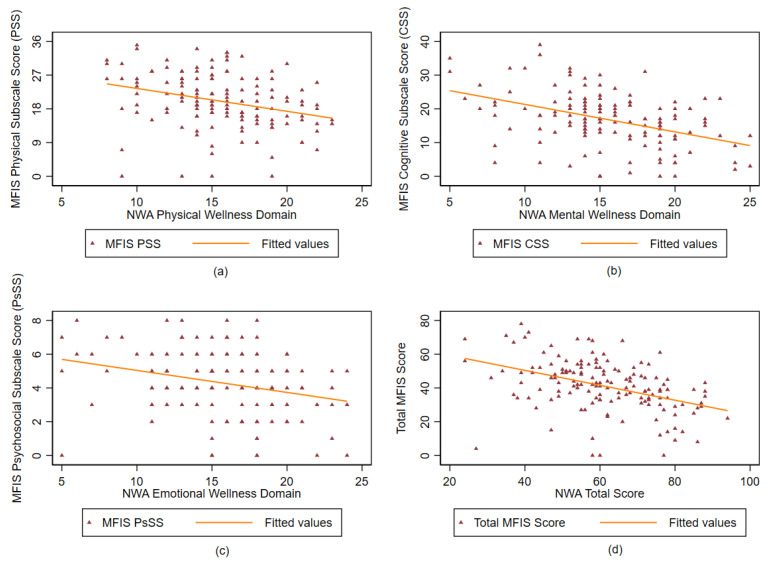
Divergent validity of National Center on Health, Physical Activity and Disability (NCHPAD) Wellness Assessment (NWA) tool (N = 149): (**a**) Modified Fatigue Impact Scale (MFIS) physical subscale scores plotted against NWA physical wellness domain scores; (**b**) MFIS cognitive subscale scores plotted against NWA mental wellness scores; (**c**) MFIS psychosocial subscale scores plotted against NWA emotional/spiritual wellness domain scores; (**d**) Total MFIS scores plotted against NWA total scores.

NWA Overall and Total Score: NWA overall and total wellness scores had weak (ranged between −0.44 and −0.11) and significant correlations with all MFIS items, except for the MFIS psychosocial subscale item (item 9) on being limited in the ability to do things away from home. These NWA scores showed weak correlations (ranging from −0.42 to −0.32 for overall wellness and −0.47 to −0.32 for total wellness, *p* < 0.001) with all MFIS composite scores. Figure 2 shows the domain-level correlations of NWA with MFIS.

### 3.4. Confirmatory Factor Analysis

**Figure 3:**
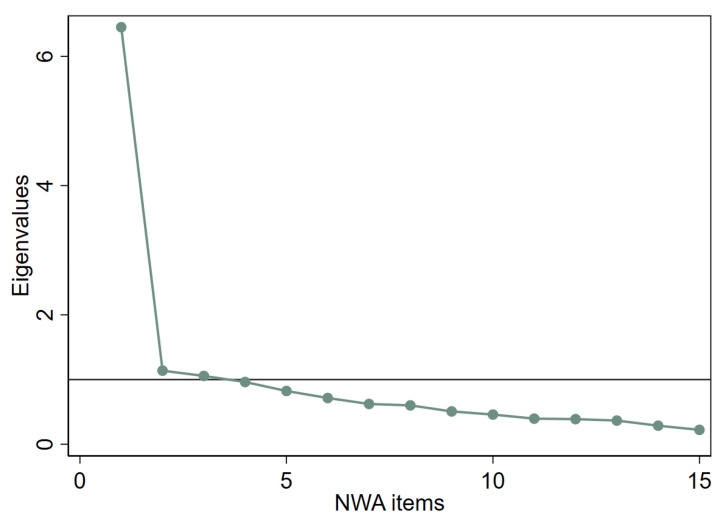
Scree plot of eigenvalues. The figure shows eigenvalues from the factor analysis of National Center on Health, Physical Activity and Disability (NCHPAD) Wellness Assessment (NWA), with three factors showing eigenvalue greater than 1.

Factor analysis of 180 observations using oblique promax rotation confirmed the appropriateness of a three-factor model (see Figure 3 for scree plot).

**Figure 4:**
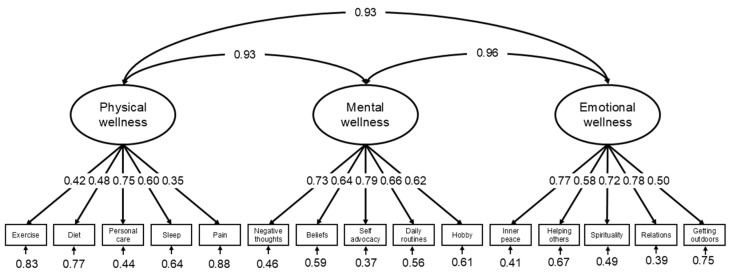
Confirmatory factor analysis of the National Center on Health, Physical Activity and Disability (NCHPAD) Wellness Assessment (NWA) tool. The figure showed factors loadings from confirmatory factor analysis of the NWA tool. The pain and exercise items within the physical wellness domain showed the least factor loadings.

Correlations between physical and mental wellness, physical and emotional/spiritual wellness and mental and emotional/spiritual wellness factors were 0.93, 0.93 and 0.96, respectively (*p* < 0.001 for all). Factor loadings for all items within each factor were statistically significant (*p* < 0.001 for all). The path diagram for the model with the original item compositions is shown in Figure 4.

Considering the high inter-factorial correlations, we also explored a higher-order model to understand whether NWA captured a broader general wellness construct. However, we observed that model fit indices were better in the original three-factor model. Fit indices for the original model showed following estimates: χ^2^(87) = 198.57 (*p* < 0.05), CFI: 0.90, RMSEA: 0.09 (pclose < 0.05), AIC: 7148.48 and BIC: 7301.74. Fit indices showed slightly improved estimates for a model excluding two items in the physical wellness domain with factor loadings <0.45 [31] (i.e., satisfaction with managing physical pain (factor loading 0.35) and regular physical exercise (factor loading 0.42)). The highest modification indices were observed between inner peace and spiritual practice items within emotional/spiritual wellness domain (16.84, *p* < 0.05), as well as between managing negative thoughts and coping with negative feelings items within mental wellness domain (14.59, *p* < 0.05). Accounting for these residual covariances within mental and emotional/spiritual wellness domains in the CFA for original tool structure did not improve model fit. However, RMSEA was significantly improved (pclose = 0.34) compared to the original model when we accounted for these covariances in the CFA after excluding pain and exercise items. Fit indices for all models are presented in Table 4. Cronbach’s α for the physical, mental, and emotional/spiritual wellness factors were 0.66, 0.82 and 0.80, respectively.

## 4. Discussion

This study represents the first attempt to assess the validity of the NWA tool. Establishing a baseline assessment using a brief, validated wellness tool can provide crucial information for customizing wellness programs, particularly during transition planning. Findings from this study will guide further refinements of this tool through subsequent iterations and validity assessment efforts. Rehabilitation researchers and clinicians can then use the tool for collecting baseline data to develop personalized wellness plans that are carried forward and reassessed by health educators and coaches to continue supporting individuals after rehabilitation, ensuring sustained wellness management.

The SF-36, GLTEQ, PROMIS-10 and MFIS, all measures were reported to be valid in our population of interest that includes multiple sclerosis and other neurological disorders [19,32,33,34]. While assessing convergent validity, we found that the GLTEQ overall and health contribution scores had the highest but weak correlations with the NWA exercise item. This finding could be explained by the fact that GLTEQ is focused on physical activity [18], while NWA physical wellness domain includes other physical wellness aspects, such as diet, sleep, self-care and pain. Therefore, it is not expected that all NWA physical wellness items would strongly correlate with GLTEQ. However, the item- or domain-level NWA physical wellness scores showed weak to no correlations even with the corresponding SF-36 or PROMIS Global-10 scores. Strong correlations were expected because both SF-36 and PROMIS Global-10 include components related to physical wellness, as represented in the WoW model [15,21,35]. Based on these results, we did not find sufficient evidence to conclude that the NWA physical wellness domain had strong convergent validity.

While both NWA and SF-36 are patient-reported outcome measures, they assess different constructs within the physical wellness domain. The physical functioning domain of the SF-36 focuses on an individual’s ability to perform specific activities (e.g., vigorous: running; moderate: vacuum cleaning; climbing stairs, walking and bathing). In contrast, items included in the NWA physical wellness domain assess an individual’s level of satisfaction with health-related behaviors such as exercise, eating habits and sleep.

Because the NWA captures perceived satisfaction with wellness behaviors rather than functional capacity, responses may not align closely with measures of physical functioning. For example, an individual may report high satisfaction with their exercise habits even if their level of physical functioning is limited, particularly among individuals who have adapted their activity levels to accommodate their disability. This pattern may also reflect adaptation or “response shift,” whereby individuals with disabilities evaluate their exercise behaviors relative to their personal capabilities and goals rather than absolute levels of physical functioning [36]. Therefore, the relatively low correlations observed between the NWA physical wellness domain and measures of physical functioning may reflect differences in construct focus rather than limitations of the instrument, suggesting that these measures capture related but distinct aspects of physical health.

Importantly, the NWA was designed as a brief criterion-referenced tool intended to provide a snapshot of wellness satisfaction to help guide individualized health coaching, rather than as a norm-referenced measure of physical functioning. In this case, responses to NWA physical wellness items are more subjective compared to responses to SF-36 physical functioning items that are more objective.

The same concept may overlie the moderate correlation between NWA pain item and SF-36 pain domain. While the items within SF-36 pain domain attempts quantifying the extent to which pain interferes with daily life, NWA pain item asks satisfaction level with managing physical pain. Similarity in these two concepts may have contributed to the observed moderate correlation. However, satisfaction with pain management in daily life may not mirror the mere ability to perform certain activity with pain causing the correlation between these two items not to be strong.

Future studies need to examine whether refinements to specific items in the physical wellness domain strengthens alignment with related constructs while preserving the instrument’s focus on perceived wellness behaviors. Additional psychometric validation studies comparing the revised NWA with other established wellness instruments will help clarify its convergent validity.

In contrast to the physical wellness domain, the significant, positive, and strong correlations of the NWA mental and emotional/spiritual wellness domains with the corresponding SF-36 and PROMIS Global-10 scores suggested that these NWA domains had strong convergent validity when compared to the corresponding scales. Similar concepts on the subjective versus objective nature of items may apply here. For instance, while the SF-36 physical functioning or pain domain attempts objective quantification of physical activities, its emotional well-being domain explores perceptions or feelings of emotional aspects (happiness, peace, etc.) of mind which is more subjective. NWA emotional/spiritual wellness items explore these aspects in terms of satisfaction which is also subjective and this similarity in nature of exploration may have contributed to its observed strong correlation with SF-36 emotional well-being domain.

The weak correlations between NWA and MFIS scores indicated that the NWA tool had strong divergent validity when compared with the MFIS. Their significant negative correlations suggested that wellness and fatigue may exist on opposite ends of a shared health continuum. The weak correlations between NWA (other than the exercise item) and GLTEQ scores further reinforced the argument that the NWA had strong divergent validity.

Although the scree plot suggested a three-factor NWA model, the CFA showed high correlations among the three factors which may suggest redundancy of factors. In this case, our interpretation of a three-factor model is based more on theoretical underpinnings than statistical correlations. There is ample scientific evidence that physical, mental and emotional well-being are interconnected, and improvement or deterioration in one impacts other aspects of well-being [37,38,39]. Despite being interconnected, available validated wellness assessment tools (e.g., WEL, PWS) have consistently identified physical, mental/intellectual and emotional dimensions as separate constructs, implying that there are subtle behavioral distinctions for measuring these constructs [4,9]. While the goal of developing NWA was brevity, further collapsing the factors based on high inter-factorial correlations may risk obscuring behaviorally relevant distinctions while measuring wellness. Given the theoretical concept, scree plot finding and good model fit indicated by CFI, the three-factor solution for NWA deemed appropriate where the tool houses multidimensional constructs with correlated components.

The CFA showed good factor loadings for items in the mental and emotional/spiritual wellness domains. While the CFI indicated a good model fit for the original model, other indices suggested a poor fit. The model fit improved modestly after excluding the pain and exercise items which had the lowest factor loadings, and both items were in the NWA physical wellness domain. The internal consistency reliability for the physical wellness domain was also poor. Based on our findings, we recommend revising the physical wellness items of the NWA tool and conducting re-assessment of its validity with the revised versions before its adoption in future studies. This iterative process should continue until the tool demonstrates strong psychometric properties.

The modification indices showed the highest residual covariances of items within the mental and emotional/spiritual wellness domains. We accounted for these covariances because of the items’ similar wording and shared content within the corresponding domains. However, accounting for these covariances resulted in an improvement in model fit only after removing the pain and exercise items from the original model. This observation further supports the fact that although pain and exercise are key components of physical wellness domains, these two items need to be further explored or rephrased to fit within the physical wellness domain of NWA to acquire strong psychometric property.

The study used existing data for secondary analysis leading to several limitations. First, our sample had female predominance that may have introduced a demographic bias to the results limiting generalizability to male participants. However, our sample reflected the natural enrollment pattern in the MENTOR program where participation was voluntary, and the MENTOR team did not have control over the sex distribution of the participants. Second, the comparators used for assessing the construct validity of NWA were not specifically included in MENTOR to assess its validity. Rather, we used the valid survey instruments included in MENTOR that seemed most appropriate for comparison. For instance, we used MFIS, which measures fatigue to assess the divergent validity of NWA, while fatigue has some degree of correlation with mental and emotional wellness. However, the items measuring mental or emotional/spiritual wellness within NWA focus on psychological flourishing, while MFIS focuses on functional implications of fatigue. Therefore, MFIS was selected as the most theoretically justifiable measure from the existing dataset to assess divergent validity. Third, although theory and our statistical findings in this effort supported a three-factor structure compared to an alternative higher order factor structure, we acknowledge that there may be concerns about structural distinctions among the factors given their high inter-factorial correlations. Fourth, our results suggested that physical wellness domain of NWA requires further refinement as indicated by lack of evidence towards strong convergent validity, least factor loadings of key physical wellness items (i.e., pain and exercise) in CFA and poor internal consistency reliability. Future efforts need to be rigorously designed with specific goals to restructure NWA (with special attention to pain and exercise items) based on the results of this study and compare the psychometric properties of physical wellness domain with other validated wellness instruments. Additionally, we could not assess concurrent validity, as this would require comparing NWA with a gold standard measure. Future MENTOR program could include WEL which is a gold standard wellness measurement [3], ideally over one intervention iteration, to facilitate a robust assessment of NWA’s construct and concurrent validity. We also plan to evaluate NWA for content validity and test-retest reliability as an immediate future step, which could not be conducted in the current effort due to limitation on retrospective analysis using existing data. Finally, multiple imputation could have been an alternative approach to address missingness, but statistical tests indicated that missingness was completely at random in our data. When data are missing completely at random, both multiple imputation and complete-case analysis have negligible bias [40]. Therefore, we chose to use complete-case analysis to preserve model transparency and interpretability given the consistency of results in two different MENTOR cohorts.

## 5. Conclusions

The current version of the 16-item NWA tool designed to assess wellness of individuals with PD showed strong convergent validity for mental and emotional/spiritual domains, strong divergent validity for all domains, with CFA showing evidence supporting a three-factor model. Given the lack of evidence on strong convergent validity of the physical wellness domain, our future research will emphasize revisiting the NWA physical wellness items followed by validity reassessments until the tool obtains strong psychometric properties for all domains.

## Figures and Tables

**Table 1:** Participant demographics (N = 180).

Variables		
**Age (years), mean ± SD**		51.76 ± 14.67
**Sex, *n* (%)**		
	Male	56 (31.11)
	Female	123 (68.33)
	Missing	1 (0.56)
**Race, *n* (%)**		
	Black/African American	44 (24.44)
	White/Caucasian	111 (61.67)
	Other	25 (13.89)
**Primary disability, *n* (%)**		
	Multiple sclerosis	24 (13.33)
	Brain related	43 (23.89)
	Spinal cord related	35 (19.44)
	Multiple	40 (22.22)
	Other	38 (21.11)

**Table 2:** Descriptive statistics of the NWA scores (N = 180).

Domain	Items (Over the Past Week, Satisfaction Related to…)	Mean ± SD	Median	IQR (Percentile Scores: 25%, 75%)
**Physical Wellness**				
	1. Regular physical exercise	2.74 ± 1.12	3	2 (2, 4)
	2. Eating healthy diet	3.01 ± 1.00	3	2 (2, 4)
	3. Taking care of personal needs	3.86 ± 1.15	4	2 (3, 5)
	4. Good night’s sleep	3.01 ± 1.05	3	2 (2, 4)
	5. Managing physical pain affecting daily activities	2.77 ± 1.05	3	1 (2, 3)
**Domain score**		15.38 ± 3.49	15	5 (13, 18)
**Mental Wellness**				
	1. Managing negative thoughts	3.01 ± 1.12	3	2 (2, 4)
	2. Following core values	3.12 ± 1.03	3	2 (2, 4)
	3. Self-advocating	3.34 ± 1.07	3	1 (3, 4)
	4. Managing negative feelings affecting daily routines	3.09 ± 1.04	3	2 (2, 4)
	5. Participating in a regular hobby	3.1 ± 1.20	3	2 (2, 4)
**Domain score**		15.66 ± 4.14	15	5 (14, 19)
**Emotional/Spiritual Wellness**				
	1. Having inner peace	3.23 ± 1.08	3	1 (3, 4)
	2. Helping others	2.94 ± 1.09	3	2 (2, 4)
	3. Valuing spiritual practice	3.34 ± 0.98	3	1 (3, 4)
	4. Handling challenging relationships	3.38 ± 1.06	4	1 (3, 4)
	5. Getting outdoors	2.94 ± 1.11	3	2 (2, 4)
**Domain score**		15.84 ± 3.96	16	5 (13, 18)
**Overall Wellness Score**		14.14 ± 4.75	15	5 (10, 15)
**Total NWA Score**		61.02 ± 14.11	60	19 (53, 72)

NWA: National Center on Health, Physical Activity and Disability (NCHPAD) Wellness Assessment; SD: Standard Deviation; IQR: Interquartile Range.

**Table 3:** Spearman correlation between SF-36 domains and NWA items and domains (N = 149).

NWA	SF-36 Physical Functioning	SF-36 Pain	SF-36 Physical Component Score	SF-36 Mental Component Score	SF-36 Emotional Well-Being
**Physical wellness domain,** correlation (*p*-value)					
Item 1	0.02 (0.79)	0.04 (0.65)	−0.04 (0.67)	0.37 * (<0.001)	0.29 * (<0.001)
Item 2	−0.01 (0.92)	0.10 (0.22)	−0.09 (0.30)	0.31 * (<0.001)	0.33 * (<0.001)
Item 3	−0.07 (0.36)	0.15 (0.08)	−0.09 (0.30)	0.36 * (<0.001)	0.39 * (<0.001)
Item 4	−0.05 (0.58)	0.28 * (<0.001)	0.003 (0.98)	0.40 * (<0.001)	0.38 * (<0.001)
Item 5	−0.08 (0.33)	0.43 * (<0.001)	0.05 (0.51)	0.31 * (<0.001)	0.34 * (<0.001)
Domain score	−0.07 (0.43)	0.29 * (<0.001)	−0.06 (0.45)	0.55 * (<0.001)	0.54 * (<0.001)
**Mental wellness domain,** correlation (*p*-value)					
Item 6	−0.21 ^†^ (0.01)	0.22 ^†^ (0.01)	−0.22 ^†^ (0.006)	0.53 * (<0.001)	0.56 * (<0.001)
Item 7	−0.08 (0.35)	0.20 ^†^ (0.01)	−0.07 (0.39)	0.43 * (<0.001)	0.36 * (<0.001)
Item 8	−0.10 (0.21)	0.18 ^†^ (0.03)	−0.13 (0.13)	0.47 * (<0.001)	0.43 * (<0.001)
Item 9	−0.03 (0.73)	0.24 ^†^ (0.003)	−0.03 (0.73)	0.47 * (<0.001)	0.44 * (<0.001)
Item 10	−0.03 (0.76)	0.21 ^†^ (0.01)	0.04 (0.66)	0.38 * (<0.001)	0.44 * (<0.001)
Domain score	−0.13 (0.11)	0.27 * (<0.001)	−0.12 (0.14)	0.61 * (<0.001)	0.59 * (<0.001)
**Emotional/spiritual wellness domain,** correlation (*p*-value)					
Item 11	−0.10 (0.24)	0.16 (0.05)	−0.13 (0.10)	0.44 * (<0.001)	0.56 * (<0.001)
Item 12	0.05 (0.57)	0.11 (0.18)	0.001 (0.99)	0.41 * (<0.001)	0.44 * (<0.001)
Item 13	−0.10 (0.23)	0.14 (0.08)	−0.12 (0.14)	0.38 * (<0.001)	0.50 * (<0.001)
Item 14	−0.18 ^†^ (0.03)	0.24 ^†^ (0.003)	−0.11 (0.17)	0.49 * (<0.001)	0.47 * (<0.001)
Item 15	0.06 (0.49)	0.16 (0.05)	0.04 (0.67)	0.29 * (<0.001)	0.30 * (<0.001)
Domain score	−0.05 (0.55)	0.20 ^†^ (0.01)	−0.08 (0.35)	0.53 * (<0.001)	0.61 * (<0.001)
Item 16	0.01 (0.91)	0.22 ^†^ (0.01)	0.02 (0.80)	0.50 * (<0.001)	0.59 * (<0.001)
Total NWA score	−0.06 (0.47)	0.28 * (<0.001)	−0.06 (0.50)	0.62 * (<0.001)	0.66 * (<0.001)

* *p* < 0.001, ^†^ *p* < 0.05. NWA: National Center on Health, Physical Activity and Disability (NCHPAD) Wellness Assessment; SF-36: Short Form-36 Health Survey.

**Table 4:** Model fit statistics from confirmatory factor analysis of NWA tool (N = 180).

	Model 1	Model 2	Model 3	Model 4	Model 5
**Chi-square statistic (*p*-value)**	198.57 (<0.05)	165.76 (<0.05)	124.06 (<0.05)	92.20 (<0.05)	204.48 (<0.05)
**CFI**	0.90	0.91	0.94	0.97	0.89
**RMSEA**	0.09	0.08	0.08	0.06	0.08
**AIC**	7148.48	6636.06	6109.84	6081.98	7148.38
**BIC**	7301.74	6779.74	6243.94	6222.47	7292.06

NWA: National Center on Health, Physical Activity and Disability (NCHPAD) Wellness Assessment (NWA); CFI: Comparative Fit Index; RMSEA: Root Mean Square Error of Approximation; AIC: Akaike Information Criterion; BIC: Bayesian Information Criterion; Model 1: Original tool; Model 2: Excluding item 5; Model 3: Excluding items 5 and 1, Model 4: Accounting for residual covariance in model 3; Model 5: One-factor model considering high inter-factorial correlations in three-factor models.

## Data Availability

The raw data supporting the conclusions of this article will be made available by the authors on reasonable request. Data is protected due to privacy concerns of the patients.

## Supplementary Materials

The following supporting information can be downloaded at: https://www.mdpi.com/article/10.3390/healthcare14081074/s1, Table S1: Spearman correlations between NWA physical wellness and SF-36 physical functioning at item level; Table S2: Spearman correlations between NWA physical wellness and SF-36 pain at item level; Table S3: Spearman correlations between NWA emotional/spiritual wellness and SF-36 emotional well-being at item level; Table S4: Patient demographics (N = 1498) in the extended analysis; Table S5: Descriptive statistics of the NWA and PROMIS Global-10 scores (N = 1498) in the extended analysis; Table S6: Spearman correlations between NWA and PROMIS Global-10 scores (N = 1498) in the extended analysis.

## Abbreviations

The following abbreviations are used in this manuscript:

AIC	Akaike information criterion
BIC	Bayesian information criterion
BMS-WBCI	Body–mind–spirit wellness behavior and characteristic inventory
CFA	Confirmatory factor analysis
CFI	Comparative fit index
GLTEQ	Godin Leisure-Time Exercise Questionnaire
LWA	Lakeshore Wellness Assessment
MCS	Mental component score
MENTOR	Mindfulness, Exercise and Nutrition to Optimize Resilience
MFIS	Modified Fatigue Impact Scale
MLE	Maximum likelihood estimation
NCHPAD	National Center on Health, Physical Activity and Disability
NWA	NCHPAD Wellness Assessment
OLP	Optimal living profile
PCS	Physical component score
PD	Physical disability
PROMIS	Patient-Reported Outcomes Measurement Information System
PWS	Perceived Wellness Survey
RMSEA	Root mean square error of approximation
SF-36	36-Item Short Form Health Survey
UAB	University of Alabama at Birmingham
WEL	Wellness Evaluation of Lifestyle
WoW	Wheel of Wellness

## Appendix A

### Appendix A.1

This section shows the descriptive statistics of SF-36, GLTEQ and MFIS.

**Table A1:** Descriptive statistics of SF-36, GLTEQ and MFIS.

SF-36 Domains (n = 149)	Mean ± SD	Median	IQR (Percentile Scores: 25%, 75%
Physical functioning	37.65 ± 29.20	35	50 (10, 60)
Role limitations due to physical health	29.03 ± 33.40	25	50 (0, 50)
Role limitations due to emotional problems	42.73 ± 40.10	33.33	100 (0, 100)
Energy/fatigue	44.53 ± 19.77	45	30 (30, 60)
Emotional well-being	64.03 ± 18.10	64	28 (52, 80)
Social functioning	56.80 ± 25.89	62.5	37.5 (37.5, 75)
Pain	53.02 ± 25.14	60	40 (40, 80)
General health	44.13 ± 14.94	45	25 (30, 55)
Physical component score	34.12 ± 9.26	33.48	13.79 (26.84, 40.63)
Mental component score	41.07 ± 13.87	39.74	20.24 (31.26, 51.50)
**GLTEQ**			
GLTEQ overall score (n = 172)	69.63 ± 94.50	36	70 (13, 83)
GLTEQ health contribution score (n = 174)	46.14 ± 68.36	24.5	50 (0, 50)
**MFIS**			
Physical subscale	20.07 ± 7.19	20	10 (16, 26)
Cognitive subscale	16.74 ± 7.97	17	9 (12, 21)
Psychosocial subscale	4.29 ± 1.87	4	3 (3, 6)
Total MFIS score	41.11 ± 15.20	42	17 (33, 50)

SF-36: Short Form-36 Health Survey; GLTEQ: Godin Leisure-Time Exercise Questionnaire; MFIS: Modified Fatigue Impact Scale; SD: Standard Deviation; IQR: Interquartile Range.

### Appendix A.2

This section shows the Spearman correlations between NWA and GLTEQ.

**Table A2:** Spearman correlation between NWA physical wellness and GLTEQ.

NWA		GLTEQ Overall Score (n = 172)	GLTEQ Health Contribution Score (n = 174)
**Physical Wellness**			
	1. Regular physical exercise	0.31 (<0.001)	0.40 (<0.001)
	2. Eating healthy diet	0.29 (<0.001)	0.27 (<0.001)
	3. Taking care of personal needs	0.18 (0.02)	0.18 (0.02)
	4. Good night’s sleep	0.12 (0.11)	0.16 (0.04)
	5. Managing physical pain affecting daily activities	−0.01 (0.86)	0.01 (0.95)
Domain score		0.26 (<0.001)	0.30 (<0.001)
Overall wellness score		0.19 (0.01)	0.22 (0.003)
Total NWA score		0.26 (<0.001)	0.29 (<0.001)

NWA: National Center on Health, Physical Activity and Disability (NCHPAD) Wellness Assessment; GLTEQ: Godin Leisure-Time Exercise Questionnaire.
