# Supplementary material for: Construct Validity and Confirmatory Factor Analysis of the National Center on Health, Physical Activity and Disability Wellness Assessment Tool
Source: Healthcare (Basel). 2026 Apr 17;14(8):1074. doi: 10.3390/healthcare14081074 (PMC13116839; doi:10.3390/healthcare14081074)
Supplement: Supplementary file 1 [file healthcare-14-01074-s001.zip › Table S6.pdf]

Table S6. Spearman correlations between NWA and PROMIS Global-10 scores (N=1498) in the extended analysis.

NWA		PROMIS Global physical health T-score	PROMIS Global mental health T-score
**Domain**	**Items (Over the past week, satisfaction related to…)**		
Physical Wellness			
	1. Regular physical exercise	0.35 (<0.001)	0.27 (<0.001)
	2. Eating healthy diet	0.23 (<0.001)	0.27 (<0.001)
	3. Taking care of personal needs	0.26 (<0.001)	0.31 (<0.001)
	4. Good night's sleep	0.33 (<0.001)	0.31 (<0.001)
	5. Managing physical pain affecting daily activities	0.39 (<0.001)	0.27 (<0.001)
Domain score		0.45 (<0.001)	0.42 (<0.001)
Mental Wellness			
	1. Managing negative thoughts	0.28 (<0.001)	0.55 (<0.001)
	2. Following core values	0.34 (<0.001)	0.47 (<0.001)
	3. Self-advocating	0.28 (<0.001)	0.44 (<0.001)
	4. Managing negative feelings affecting daily routines	0.33 (<0.001)	0.52 (<0.001)
	5. Participating in a regular hobby	0.31 (<0.001)	0.41 (<0.001)
Domain score		0.39 (<0.001)	0.60 (<0.001)
NWA: National Center on Health, Physical Activity and Disability (NCHPAD) Wellness Assessment; PROMIS: Patient-Reported Outcomes Measurement Information System			
