# Supplementary material for: Construct Validity and Confirmatory Factor Analysis of the National Center on Health, Physical Activity and Disability Wellness Assessment Tool
Source: Healthcare (Basel). 2026 Apr 17;14(8):1074. doi: 10.3390/healthcare14081074 (PMC13116839; doi:10.3390/healthcare14081074)
Supplement: Supplementary file 1 [file healthcare-14-01074-s001.zip › Table S2.pdf]

Table S2. Spearman Correlation analysis between NWA physical wellness domain and SF-36 Pain domain at item level.

	SF-36	
NWA	**Item 21**	**Pain domain**
**1.Getting enough exercise**	0.04 (0.65)	0.04 (0.65)
**2.Eating healthy diet**	0.10 (0.22)	0.10 (0.22)
**3.Taking care of personal needs**	0.15 (0.08)	0.15 (0.08)
**4.Getting good sleep**	0.28 (<0.001)	0.28 (<0.001)
**5.Managing physical pain**	0.43 (<0.001)	0.43 (<0.001)
**Physical wellness domain**	0.29 (<0.001)	0.29 (<0.001)
**Mental wellness domain**	0.27 (<0.001)	0.27 (<0.001)
**Emotional wellness domain**	0.20 (0.01)	0.20 (0.01)
**Overall wellness**	0.22 (0.01)	0.22 (0.01)
**Total wellness**	0.28 (<0.001)	0.28 (<0.001)
NWA: National Center on Health, Physical Activity and Disability (NCHPAD) Wellness Assessment; SF-36: Short Form-36 Health Survey		
