# Supplementary material for: Construct Validity and Confirmatory Factor Analysis of the National Center on Health, Physical Activity and Disability Wellness Assessment Tool
Source: Healthcare (Basel). 2026 Apr 17;14(8):1074. doi: 10.3390/healthcare14081074 (PMC13116839; doi:10.3390/healthcare14081074)
Supplement: Supplementary file 1 [file healthcare-14-01074-s001.zip › Table S3.pdf]

Table S3. Spearman correlations between NWA emotional/spiritual wellness and SF-36 emotional well-being at item level.

	SF-36					
**NWA**	**Item 24**	**Item 25**	**Item 26**	**Item 28**	**Item 30**	**Emotional well-being domain**
**11.Inner peace**	0.26 (0.001)	0.46 (<0.001)	0.50 (<0.001)	0.46 (<0.001)	0.42 (<0.001)	0.56 (<0.001)
**12.Imp contribution**	0.24 (0.003)	0.31 (<0.001)	0.36 (<0.001)	0.44 (<0.001)	0.30 (<0.001)	0.44 (<0.001)
**13.Spiritual practice**	0.26 (0.001)	0.44 (<0.001)	0.40 (<0.001)	0.46 (<0.001)	0.38 (<0.001)	0.50 (<0.001)
**14.Chall relation**	0.21 (0.01)	0.42 (<0.001)	0.38 (<0.001)	0.46 (<0.001)	0.35 (<0.001)	0.47 (<0.001)
**15.Get outdoor**	0.25 (0.003)	0.18 (0.03)	0.24 (0.003)	0.27 (0.001)	0.21 (0.01)	0.30 (<0.001)
**Overall wellness**	0.29 (<0.001)	0.46 (<0.001)	0.46 (<0.001)	0.52 (<0.001)	0.47 (<0.001)	0.59 (<0.001)
**Total wellness**	0.33 (<0.001)	0.53 (<0.001)	0.53 (<0.001)	0.58 (<0.001)	0.51 (<0.001)	0.66 (<0.001)

NWA: National Center on Health, Physical Activity and Disability (NCHPAD) Wellness Assessment; SF-36: Short Form-36 Health Survey
