# Supplementary material for: Construct Validity and Confirmatory Factor Analysis of the National Center on Health, Physical Activity and Disability Wellness Assessment Tool
Source: Healthcare (Basel). 2026 Apr 17;14(8):1074. doi: 10.3390/healthcare14081074 (PMC13116839; doi:10.3390/healthcare14081074)
Supplement: Supplementary file 1 [file healthcare-14-01074-s001.zip › Table S4.pdf]

Table S4. Patient demographics (N=1498) in the extended analysis

		**Mean (±SD)/Frequency (%)**
Age		55.67 (±13.97)
Sex		
	Female	940 (62.75)
	Male	557 (37.18)
	None of these describe me	1 (0.07)
Race		
	Black or African American	464 (30.97)
	White	794 (53.00)
	Other	240 (16.02)
Primary disability		
	Brain related	842 (56.21)
	Spinal cord related	114 (7.61)
	Multiple	353 (23.56)
	Others	189 (12.62)
