# Supplementary material for: Construct Validity and Confirmatory Factor Analysis of the National Center on Health, Physical Activity and Disability Wellness Assessment Tool
Source: Healthcare (Basel). 2026 Apr 17;14(8):1074. doi: 10.3390/healthcare14081074 (PMC13116839; doi:10.3390/healthcare14081074)
Supplement: Supplementary file 1 [file healthcare-14-01074-s001.zip › Table S5.pdf]

**Table S5.** Descriptive statistics of the NWA and PROMIS Global-10 scores (N=1498) in the extended analysis.

| **NWA** | | | | |
|---|---|---|---|---|
| **Domain** | **Items (Over the past week, satisfaction related to…)** | **Mean ± SD** | **Median** | **IQR (Percentile scores: 25%, 75%)** |
| **Physical Wellness** | | | | |
| | 1. Regular physical exercise | 2.51 (±1.12) | 2 | 1 (2, 3) |
| | 2. Eating healthy diet | 2.99 (±1.12) | 3 | 2 (2, 4) |
| | 3. Taking care of personal needs | 3.73 (±1.15) | 4 | 2 (3, 5) |
| | 4. Good night's sleep | 3.08 (±1.24) | 3 | 2 (2, 4) |
| | 5. Managing physical pain affecting daily activities | 2.90 (±1.10) | 3 | 2 (2, 4) |
| Domain score | | 15.22 (±4.02) | 15 | 6 (12, 18) |
| **Mental Wellness** | | | | |
| | 1. Managing negative thoughts | 3.32 (±1.10) | 3 | 1 (3, 4) |
| | 2. Following core values | 3.26 (±1.09) | 3 | 2 (2, 4) |
| | 3. Self-advocating | 3.41 (±1.16) | 4 | 1 (3, 4) |
| | 4. Managing negative feelings affecting daily routines | 3.27 (±1.10) | 3 | 2 (2, 4) |
| | 5. Participating in a regular hobby | 2.98 (±1.23) | 3 | 2 (2, 4) |
| Domain score | | 16.24 (±4.53) | 16 | 7 (13, 20) |
| **Emotional/Spiritual Wellness** | | | | |
| | 1. Having inner peace | 3.41 (±1.16) | 4 | 1 (3, 4) |
| | 2. Helping others | 3.13 (±1.18) | 3 | 2 (2, 4) |
| | 3. Valuing spiritual practice | 3.71 (±1.10) | 4 | 2 (3, 5) |
| | 4. Handling challenging relationships | 3.33 (±1.10) | 3 | 1 (3, 4) |
| | 5. Getting outdoors | 2.86 (±1.21) | 3 | 2 (2, 4) |
| Domain score | | 16.43 (±4.45) | 16.5 | 6 (14, 20) |
| Overall Wellness | | 13.91 (±5.41) | 15 | 10 (10, 20) |
| Total NWA Score | | 61.81 (±15.33) | 62 | 22 (51, 73) |
| **PROMIS Global-10** | | | | |
| PROMIS Global physical health T-score | | 38.81 (±7.40) | 37.4 | 7.4 (34.9, 42.3) |
| PROMIS Global mental health T-score | | 42.93 (±9.07) | 41.1 | 12 (36.3, 48.3) |

NWA: National Center on Health, Physical Activity and Disability (NCHPAD) Wellness Assessment; PROMIS: Patient-Reported Outcomes Measurement Information System; SD: Standard Deviation; IQR: Interquartile Range
