# Supplementary material for: Construct Validity and Confirmatory Factor Analysis of the National Center on Health, Physical Activity and Disability Wellness Assessment Tool
Source: Healthcare (Basel). 2026 Apr 17;14(8):1074. doi: 10.3390/healthcare14081074 (PMC13116839; doi:10.3390/healthcare14081074)
Supplement: Supplementary file 1 [file Table S1.pdf › Table S1.pdf]

Table S1. Spearman correlations between NWA physical wellness and SF-36 physical functioning at item level.

	SF-36									
**NWA**	**Item 3**	**Item 4**	**Item 5**	**Item 6**	**Item 7**	**Item 8**	**Item 9**	**Item 10**	**Item 11**	**Item 12**	**Physical func. domain**
**1.Getting enough exercise**	0.07 (0.42)	0.04 (0.62)	-0.06 (0.50)	0.11 (0.19)	0.01 (0.92)	-0.12 (0.15)	0.02 (0.82)	0.06 (0.46)	0.07 (0.38)	-0.03 (0.72)	0.02 (0.79)
**2.Eating healthy diet**	-0.08 (0.34)	0.02 (0.76)	-0.01 (0.91)	0.09 (0.27)	0.005 (0.96)	-0.03 (0.74)	-0.08 (0.35)	-0.07 (0.43)	-0.001 (0.99)	-0.02 (0.85)	-0.01 (0.92)
**3.Taking care of personal needs**	-0.16 (0.05)	0.04 (0.61)	-0.10 (0.24)	-0.06 (0.46)	-0.05 (0.55)	-0.09 (0.30)	-0.16 (0.06)	-0.10 (0.22)	-0.03 (0.70)	0.03 (0.67)	-0.07 (0.36)
**4.Getting good sleep**	-0.07 (0.37)	-0.05 (0.58)	-0.06 (0.47)	0.02 (0.82)	-0.04 (0.60)	-0.09 (0.30)	-0.08 (0.36)	-0.08 (0.34)	0.03 (0.73)	0.002 (0.99)	-0.05 (0.58)
**5.Managing physical pain**	-0.05 (0.53)	-0.06 (0.48)	-0.05 (0.51)	0.01 (0.88)	-0.01 (0.88)	-0.10 (0.25)	-0.07 (0.38)	-0.14 (0.10)	-0.10 (0.22)	-0.13 (0.12)	-0.08 (0.33)
**Physical wellness domain**	-0.07 (0.38)	-0.02 (0.84)	-0.09 (0.26)	0.05 (0.56)	-0.03 (0.70)	-0.11 (0.17)	-0.12 (0.14)	-0.11 (0.18)	-0.02 (0.77)	-0.04 (0.66)	-0.07 (0.43)
**Mental wellness domain**	-0.03 (0.74)	-0.05 (0.57)	-0.09 (0.28)	-0.03 (0.70)	-0.10 (0.20)	-0.16 (0.06)	-0.19 (0.02)	-0.16 (0.05)	-0.12 (0.15)	-0.09 (0.30)	-0.13 (0.11)
**Emotional wellness domain**	-0.02 (0.80)	0.03 (0.73)	-0.03 (0.76)	-0.001 (0.99)	-0.10 (0.24)	-0.13 (0.13)	-0.10 (0.20)	-0.02 (0.82)	-0.04 (0.66)	0.02 (0.82)	-0.05 (0.55)
**Overall wellness**	0.01 (0.88)	0.10 (0.23)	0.08 (0.31)	0.06 (0.43)	0.01 (0.87)	-0.07 (0.38)	-0.05 (0.56)	0.001 (0.99)	0.01 (0.88)	-0.03 (0.74)	0.01 (0.91)
**Total Wellness**	-0.03 (0.75)	0.03 (0.71)	-0.02 (0.77)	0.03 (0.73)	-0.06 (0.50)	-0.14 (0.10)	-0.13 (0.12)	-0.07 (0.38)	-0.04 (0.66)	-0.04 (0.64)	-0.06 (0.47)

NWA: National Center on Health, Physical Activity and Disability (NCHPAD) Wellness Assessment; SF-36: Short Form-36 Health Survey
